# Plasma Circulating lncRNAs: MALAT1 and NEAT1 as Biomarkers of Radiation-Induced Adverse Effects in Laryngeal Cancer Patients

**DOI:** 10.3390/diagnostics15060676

**Published:** 2025-03-10

**Authors:** Marcin Mazurek, Anna Brzozowska, Teresa Małecka-Massalska, Tomasz Powrózek

**Affiliations:** 1 Department of Human Physiology of Chair of Preclinical Sciences, Medical University of Lublin, 20-059 Lublin, Poland; teresa.malecka-massalska@umlub.pl (T.M.-M.); tomaszpowrozek@gmail.com (T.P.); 2 Department of Radiotherapy, St. John of Dukla Lublin Region Cancer Center, 20-090 Lublin, Poland; annabrzo@poczta.onet.pl

**Keywords:** laryngeal cancer, lncRNAs, radiotherapy, MALAT1, NEAT1, radiotherapy adverse effects

## Abstract

**Background**: The majority of head and neck cancers (HNCs) occur in the larynx. In clinical practice, adverse effects are frequently observed in laryngeal cancer (LC) patients undergoing radiotherapy (RT). Therefore, investigating markers that can predict these unfavorable events is of interest. Long non-coding RNAs (lncRNAs) have emerged as potential biomarkers for the early identification of patients susceptible to post-RT toxicity. MALAT1 and NEAT1 regulate various cellular processes, the inflammatory response, and resistance to anti-cancer treatments; however, their impact on the portability of post-RT adverse effects remains unknown. The aim of this study was to evaluate the clinical value of two plasma-circulating lncRNAs, MALAT1 and NEAT1, as predictive biomarkers for post-RT adverse effects in LC patients. **Methods**: The expression levels of the studied lncRNAs were determined using real-time quantitative reverse transcription PCR (qRT-PCR) in plasma samples obtained from 70 LC patients before the initiation of RT. These levels were then correlated with patient outcomes. **Results**: A low expression of MALAT1 was associated with a significantly higher probability of anemia, liver failure, and severe malnutrition (OR = 5.36; *p* = 0.040, OR = 6.07; *p* = 0.037, OR = 9.75; *p* < 0.001, respectively) after the completion of RT. Similarly, patients with low NEAT1 expression had a significantly higher risk of anemia, liver failure, and mild or severe malnutrition (OR = 5.26; *p* = 0.020, OR = 5.70; *p* = 0.016, OR = 13.09; *p* = 0.002, respectively). Simultaneous lower expression levels of both lncRNAs were significantly associated with shorter median overall survival (OS) in RT-treated LC patients (HR = 5.44; *p* = 0.001). **Conclusions**: The analysis of MALAT1 and NEAT1 expression indicates clinical utility in predicting toxic events induced by RT-based therapy.

## 1. Introduction

Approximately 90% of head and neck cancers (HNCs) are squamous cell carcinomas, with 30–40% found in the larynx [1,2]. Around 60% of laryngeal cancer (LC) patients are diagnosed at advanced stages [3,4]. Treatment options for stage III/IV LC include surgery, radiotherapy (RT), and chemoradiotherapy (C-RT). However, RT-based treatments carry a high risk of adverse effects, toxicities, and disruption of laryngeal function [5,6]. Acute (early) toxicity refers to early adverse effects occurring within 2–3 weeks after the completion of RT, while late toxicity manifests from weeks to years after radiation exposure [7]. In clinical practice, nearly 50% of post-RT adverse effects are manifested as acute toxicity (grade 2 or 3), including skin reactions, dysphagia, radiation-induced oral mucositis (RIOM), and bone marrow suppression [8]. The risk of both acute and late toxicities is known to be influenced by cancer characteristics, radiation dose, and methods of administration [9]. In addition, susceptibility to acute RT toxicity depends on the clinical characteristics of patients. The prevalence of anemia is 16% in HNC cases prior to RT and increases to 32% 3–5 weeks after the first radiation course [10]. According to the literature, acute renal failure affects nearly 28% of HNC patients undergoing C-RT [11].

Nowadays, particular attention is paid to molecular diagnostics, which allows for the analysis of the expression of various genetic markers associated with susceptibility to the development of RT toxicity, helping to define its severity. For this purpose, non-coding RNAs, such as long non-coding RNAs (lncRNAs) and microRNAs (miRNAs), are considered prospective molecular biomarkers that can be investigated using liquid biopsy [12]. LncRNAs are non-coding transcripts longer than 200 nucleotides and are not translated into proteins [13]. They are often tissue- or cancer-specific and can function as competitive endogenous RNAs, influencing mRNA expression via miRNA regulation [14]. Another function of lncRNAs is the regulation of gene expression through the binding of chromatin regulatory proteins. LncRNAs interact with other RNAs and proteins, leading to both transcriptional and post-transcriptional gene regulation. Strong relationships between miRNAs and lncRNAs have been confirmed. Additionally, lncRNAs can compete with miRNAs for specific sites in the non-coding regions of mRNAs, thereby promoting gene expression [15]. Alterations in lncRNAs, which act as oncogenes or tumor suppressors, have been observed in various cancers [16]. LncRNAs are believed to play a key role in resistance to anti-cancer treatments, including RT, and show potential as diagnostic biomarkers [17]. Additionally, they influence cancer cell sensitivity to therapy by modulating mechanisms like chromatin modification, DNA repair, cell cycle control, and mRNA processing [18]. The expression of several lncRNAs, including NEAT1, HNF1A-AS, ROR, GAS8-AS1, and FAL1, is altered in HNC tissues and cell lines, making them promising molecular markers and therapeutic targets for HNC [19,20,21].

It has been found that metastasis-associated lung adenocarcinoma transcript 1 (MALAT1) is widely upregulated in several tumors, including esophageal, liver, lung, and cervical carcinoma [22]. Additionally, MALAT1 expression was significantly upregulated in laryngeal, nasopharyngeal, and oral cancers [23]. MALAT1 regulates tumor-related inflammation and promotes the proliferation, motility, and epithelial–mesenchymal transition of tumor cells [24], whereas nuclear paraspeckle assembly transcript 1 (NEAT1) is involved in the cellular response to DNA damage and targets multiple pro-inflammatory factors, such as nuclear factor-kappa B (NF-κB) and cytokines (e.g., IL-1, IL-6, and TNF-α), thereby maintaining the inflammatory response [25,26]. It should be noted that MALAT1 regulates ferroptosis and modulates drug resistance through the p53 signaling pathway in the miR-34c/Myc/MALAT1 axis [27]. For instance, upregulation of NEAT1 has been noted in lung, thyroid, pancreatic, prostate, and liver cancers [28,29]. In the context of HNC, NEAT1 was found to be overexpressed in LC tissues compared with adjacent non-cancerous samples [18]. It was postulated that NEAT1 affects LC aggressiveness by promoting metastasis and tumor growth [30]. Moreover, decreased NEAT1 is involved in promoting apoptosis and inhibiting proliferation in non-small-cell lung cancer cells by increasing the secretion of cell cycle inhibitors such as p53 and p21 [31]. Similarly to MALAT1, NEAT1 also contributes to the inflammatory response through the modulation of the NF-κB pathway [32]. It is believed that MALAT1 accelerates the production of pro-inflammatory cytokines, including TNF-α and IL-6 [24], while NEAT1 contributes to cytokine production and the formation of reactive oxygen species by activated macrophages [33]. It should be noted that studied lncRNAs modulate radioresistance, but their impact on the chance of post-RT adverse effects are unknown.

The aim of this study was to assess the relationship between both MALAT1 and NEAT1 expression and the development of adverse effects after radiation therapy for locally advanced and advanced LC. The secondary objective was to investigate the impact of the studied lncRNAs on patient survival.

## 2. Material and Methods

### 2.1. Study Group

The study group consisted of 70 patients diagnosed with locally advanced or advanced squamous-cell LC. Male patients predominated, representing 82.9% of the sample compared to 17.1% being female. All participants were diagnosed and treated with RT-based therapy at the Department of Oncology at the Medical University of Lublin between 2014 and 2017. The inclusion criteria for the study were age >18 years, both genders, histopathologically confirmed LC, and completion of RT or C-RT. Exclusion criteria included autoimmune diseases, previous or concomitant cancer, and poor performance status (ECOG ≥ 2). Based on the TNM classification (8th edition), 31.4% of patients had stage III disease and 68.6% had stage IV. Previous surgical treatment was reported in 91.4% of patients. All patients received seven courses of radical RT using intensity-modulated radiotherapy (IMRT) with a total dose of 70 Gy. Alcohol consumption was assessed using the International Statistical Classification of Diseases and Related Health Problems (ICD) scale. The following groups were distinguished among smokers: current or former smokers. A non-smoker is a person who has never smoked tobacco or a person who has smoked less than 100 cigarettes in their lifetime. The current smoker or ex-smoker is defined as an adult who has smoked at least 100 cigarettes during his life or is still smoking. Physical performance status was evaluated according to the Eastern Cooperative Oncology Group (ECOG) scale. Radiation-induced oral mucositis (RIOM) severity was assessed using the Radiation Therapy Oncology Group/European Organization for Research and Treatment of Cancer (RTOG/EORTC) scale. Renal function was evaluated by measuring serum creatinine levels and the estimated glomerular filtration rate (eGFR). Liver function was assessed by measuring serum total bilirubin and transaminase (AST, ALT) levels. Hematologic toxicity was evaluated by measuring hemoglobin levels, as well as the quantity and percentage of red blood cells. Adverse events were assessed after RT, based on CTC Version 5.0 (common toxicity criteria) [34]. Additionally, anthropometric parameters, including body mass and body mass index (BMI), were measured 24 h before and after the RT course. The nutritional status of patients was assessed using the subjective global assessment (SGA) scale [35]. According to the SGA, most patients were classified as moderately (SGA-B; 45.7%) or severely (SGA-C; 47.9%) malnourished. C-reactive protein (CRP) concentration was measured post-treatment to reflect the inflammatory condition. The study was approved by the Bioethical Commission at the Medical University of Lublin (consent number: KE-0254/64/2017). The characteristics of the study group are presented in Table 1.

### 2.2. Treatment Protocol

The IMRT-based approach was applied to all patients using the ONCOR (Siemens, Munich, Germany) linear accelerator. Doses ranging from 54 to 70 Gy (with a daily dose of 2 Gy) were administered. The advanced cancer group received a total of 70 Gy in 35 fractions targeting tumors and enlarged lymph nodes. Patients at risk of volume recurrence after surgery were exposed to radiation in 33 fractions, totaling 66 Gy. The average-risk group received 60 Gy, while the low-risk group was given 54 Gy. Elective lymph nodes were treated with either 54 or 60 Gy. In addition to IMRT, some patients underwent 1–4 cycles of CTH with cisplatin and 5-fluorouracil. Each patient received therapy on a regular schedule over a total period of seven weeks.

### 2.3. lncRNA Expression Analysis

Before the initiation of RT, 5 mL of peripheral blood was collected from all study participants. The samples were centrifuged for 15 min at 1000× *g*, and the platelet-poor plasma was collected within the next 30 min. Plasma samples were stored at −80 °C until laboratory analysis. lncRNA was purified from 200 μL of plasma using a column-based method according to the manufacturer’s instructions (miRNeasy Serum/Plasma Kit, Qiagen GmbH, Hilden, Germany). Purified lncRNAs were reverse-transcribed into complementary DNA (cDNA) using the iScript Reverse Transcription Supermix for RT-qPCR kit (Bio-Rad Laboratories, Hercules, CA, USA). Amplification was performed using the StepOnePlus device (Applied Biosystems, Foster City, CA, USA) with TaqMan Universal Mastermix (ThermoFisher Scientific, Waltham, MA, USA) and TaqMan Non-coding RNA Assay (20x) probes, namely MALAT1 (assay name: Hs00273907; ThermoFisher Scientific, Waltham, MA, USA) and NEAT1 (assay name: Hs03453535; ThermoFisher Scientific, Waltham, MA, USA). All samples were analyzed in triplicate. The expression levels of the studied lncRNAs were normalized to GAPDH as an internal control (assay name: Hs02786624; ThermoFisher Scientific, Waltham, MA, USA) using 2^−ΔΔCt^ and 2^−ΔCt^ formulae.

### 2.4. Statistical Analysis

Statistical analysis was performed using MedCalc v.15.8 software (MedCalc Software, Ostend, Belgium). *p*-values below 0.05 were considered statistically significant (*p* < 0.05). The distribution of the tested parameters was assessed using the D’Agostino–Pearson test. Comparisons of the expression levels of the studied lncRNAs based on clinical-demographic factors were analyzed using the U Mann–Whitney test. Odds ratios (ORs) with 95% confidence intervals (95% CIs) were calculated to assess the risk of RT toxicity development based on lncRNA expression levels. Multivariable analysis of the risk of RT adverse effects depending on demographic, clinical, and genetic factors was performed using logistic regression. Overall survival (OS) was evaluated using the Kaplan–Meier estimator, and the hazard ratio (HR) with a 95% CI was calculated based on lncRNA expression. Receiver operating characteristic (ROC) curves were used to determine the cut-off points and evaluate the diagnostic accuracy of the studied lncRNAs in detecting adverse effects in the study population.

## 3. Results

### 3.1. Relationship Between Expression of the lncRNAs and Tumor Staging

**Figure 1:**
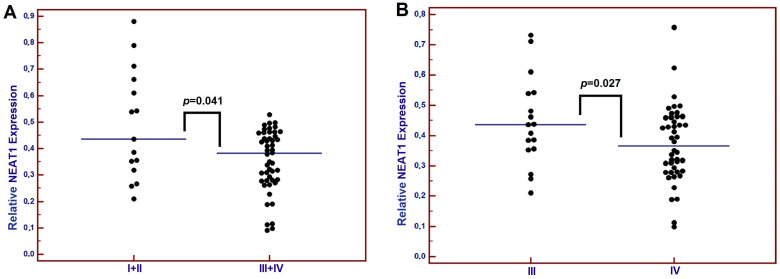
Differences in the relative expression of NEAT1 levels between groups of patients with (**A**) different tumor (T) staging; (**B**) locally advanced or advanced stages of LC according to the TNM. Abbreviations: I—T1 stage; II—T2 stage; III—T3 stage; IV—T4 stage.

Based on pre-treatment MALAT1 and NEAT1 median expression levels (median: 0.28 and 0.38, respectively), patients were divided into two groups, those with low or high lncRNA expression. In our study, 38 patients (54.3%) had a low expression and 32 patients (45.7%) had a high expression of MALAT1. For MALAT1, we found that patients with low-grade tumors (G3) had significantly lower expression of this lncRNA compared to those with higher-grade tumors (median: 0.33 vs. 0.43; *p* = 0.040). Additionally, NEAT1 expression was significantly lower in patients with T3 and T4 tumor stages compared to those with T1 and T2 stages (median: 0.38 vs. 0.43; *p* = 0.041) (Figure 1A). Of the patients, 58 (82.9%) had low and 12 (17.1%) had high NEAT1 expression. Moreover, stage IV of the disease was associated with lower NEAT1 levels (median: 0.36 vs. 0.44; *p* = 0.027) (Figure 1B). The expression of NEAT1 was significantly lower in low-grade tumors (G3) compared to high-grade tumors (G1 and G2) (median: 0.31 vs. 0.42; *p* = 0.012) (Table 2).

### 3.2. Relationship Between Expression of lncRNAs and Occurrence of Adverse Effects After RT

**Figure 2:**
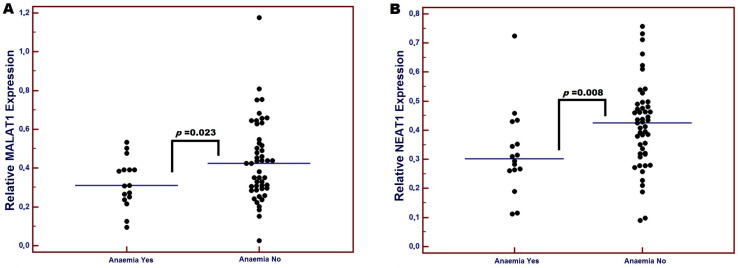
Differences in the relative expression of lncRNA depending on the presence of post-RT anemia. (**A**) MALAT1; (**B**) NEAT1.

After the completion of RT, grade 1 and 2 RIOM was found in 45/70 (64.3%) patients, while grade 3 RIOM was observed in 25/70 (35.7%) patients. Post-RT anemia was recorded in 12/70 (17.1%) patients, kidney failure in 33/70 (47.1%), and liver failure in 41/70 (58.6%) patients. In patients with post-RT anemia (hemoglobin < 12 g/dL), both low MALAT1 (median: 0.31 vs. 0.44; *p* = 0.023) and NEAT1 expression (median: 0.30 vs. 0.43; *p* = 0.008) were observed compared to those with normal hemoglobin levels (Figure 2). Additionally, patients with liver failure had a significantly lower expression of both MALAT1 (median: 0.32 vs. 0.47; *p* = 0.013) and NEAT1 (median: 0.32 vs. 0.44; *p* = 0.026). We also observed, though not significantly, higher MALAT1 (median: 0.38 vs. 0.41; *p* = 0.891) and NEAT1 (median: 0.39 vs. 0.42; *p* = 0.522) expression in patients with grade 3 RIOM. Furthermore, patients with kidney failure had an insignificantly lower expression of both MALAT1 (median: 0.32 vs. 0.40; *p* = 0.567) and NEAT1 (median: 0.34 vs. 0.40; *p* = 0.344). Interestingly, patients diagnosed with severe malnutrition according to the SGA (SGA-C) exhibited significantly lower expression levels of both MALAT1 and NEAT1 compared to well-nourished or mildly malnourished patients (SGA-A or SGA-B) (median: 0.29 vs. 0.31; *p* = 0.036 and 0.22 vs. 0.26; *p* = 0.032, respectively). A significantly lower expression of MALAT1 was observed in individuals who lost at least 5% of their body mass and in those who lost more than 10% of their initial body weight during treatment (median: 0.26 vs. 0.31; *p* = 0.032 and 0.19 vs. 0.31; *p* = 0.015, respectively). A similar significant relationship was found between NEAT1 and the presence of >10% body weight loss (median: 0.22 vs. 0.25; *p* = 0.034). LC patients with lower expression levels of both MALAT1 and NEAT1 had significantly lower BMI (<21.29 kg/m^2^) after completion of the RT (median: 0.23 vs. 0.34; *p* = 0.048 and 0.21 vs. 0.30; *p* = 0.048, respectively) (Table 3).

### 3.3. lncRNAs as Predictors of Radiation Therapy Adverse Effects

A low expression level of MALAT1 was associated with more than a 5-fold higher probability of anemia (OR = 5.36; *p* = 0.040) and over a 6-fold higher chance of liver failure (OR = 6.07; *p* = 0.037) after the completion of RT. Additionally, low MALAT1 expression was linked to nearly a 10-fold higher probability of severe malnutrition, as defined by the SGA (OR = 9.75; *p* < 0.001). A higher likelihood of body mass loss >5% (OR = 7.42; *p* < 0.001) and a lower BMI after treatment (OR = 2.84; *p* = 0.035) were also associated with low MALAT1 expression. Furthermore, patients with decreased MALAT1 expression had almost a 5-fold higher probability of increased plasma CRP concentration (>5 mg/L) after treatment (OR = 4.91; *p* = 0.002) (Table 3).

Low expression of NEAT1 was associated with more than a 5-fold higher probability of anemia (OR = 5.26; *p* = 0.020) and nearly a 6-fold higher chance of liver failure (OR = 5.70; *p* = 0.016) after the completion of RT. Additionally, LC patients with low NEAT1 expression had more than a 13-fold higher probability of mild or severe malnutrition according to the SGA (OR = 13.09; *p* = 0.002) and an 11-fold higher chance of losing >5% of their body weight after RT (OR = 11.0; *p* = 0.026). Furthermore, decreased NEAT1 expression was associated with more than a 6-fold higher probability of elevated CRP plasma concentration (>5 mg/L) in LC patients (OR = 6.15; *p* = 0.026) (Table 3).

The multivariable analysis revealed a significantly higher likelihood of grade 3 RIOM in patients undergoing RT (OR = 4.26; *p* = 0.041), those with lower MALAT1 expression (OR = 5.19; *p* = 0.021), and those with severe malnutrition according to the SGA (SGA-C) (OR = 3.46; *p* = 0.043). A lower probability of severe RIOM was associated with decreased NEAT1 expression (OR = 0.12; *p* = 0.032). A low MALAT1 expression (OR = 9.33; *p* = 0.006) and RT (OR = 16.71; *p* = 0.006) were associated with a higher probability of anemia. LC patients with poor performance status (ECOG) (OR = 4.62; *p* = 0.047), as well as lower MALAT1 expression (OR = 1.49; *p* = 0.015) and NEAT1 expression (OR = 9.09; *p* = 0.005), had a significantly greater likelihood of liver failure. Furthermore, patients undergoing RT (OR = 4.95; *p* = 0.017), those with severe malnutrition according to the SGA (SGA-C) (OR = 6.25; *p* = 0.046), and those with decreased MALAT1 levels (OR = 4.28; *p* = 0.025) had a significantly higher chance of losing 10% of their body weight.

### 3.4. Diagnostic Value of lncRNAs: MALAT1 and NEAT1 for Post-RT Adverse Events

**Figure 3:**
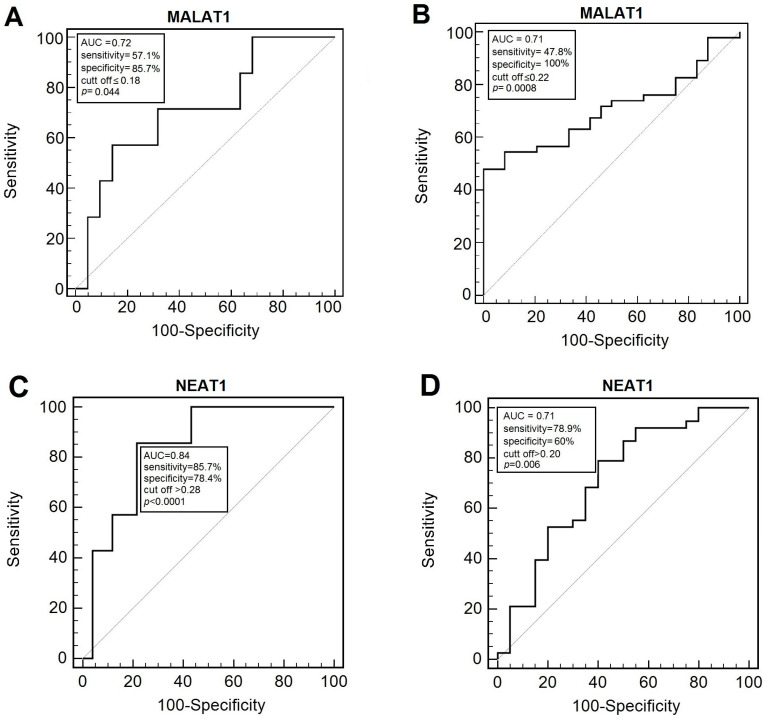
ROC curves demonstrating diagnostic accuracy of studied lncRNA expression values for discrimination of (**A**) weight loss 10%; (**B**) BMI after RT; (**C**) anemia; (**D**) liver failure.

The assessment of MALAT1 expression presented diagnostic usefulness in distinguishing between patients that lost more than 10% of their body weight during treatment with 57.1% sensitivity and 85.7% specificity (AUC = 0.72; cut-off ≤ 0.18; *p* < 0.044) (Figure 3A). Moreover, the pretreatment expression level of MALAT1 allowed us to discriminate patients with a low BMI after complete of RT with 47.85% sensitivity and 100% specificity (AUC = 0.71; cut-off ≤ 0.22; *p* < 0.0008) (Figure 3B).

Only NEAT1 demonstrated diagnostic accuracy in distinguishing between patients based on the presence of anemia, with 85.71% sensitivity and 78.43% specificity (AUC = 0.84; cut-off > 0.28; *p* < 0.001) (Figure 3C). Additionally, NEAT1 was effective in discriminating between patients with liver failure, showing 78.95% sensitivity and 60% specificity (AUC = 0.71; cut-off > 0.20; *p* < 0.006) (Figure 3D). A combined analysis of NEAT1 and MALAT1 did not improve the diagnostic accuracy of the test.

### 3.5. Relationship Between the Expression Levels of the Studied lncRNAs and Overall Survival in LC Patients

**Figure 4:**
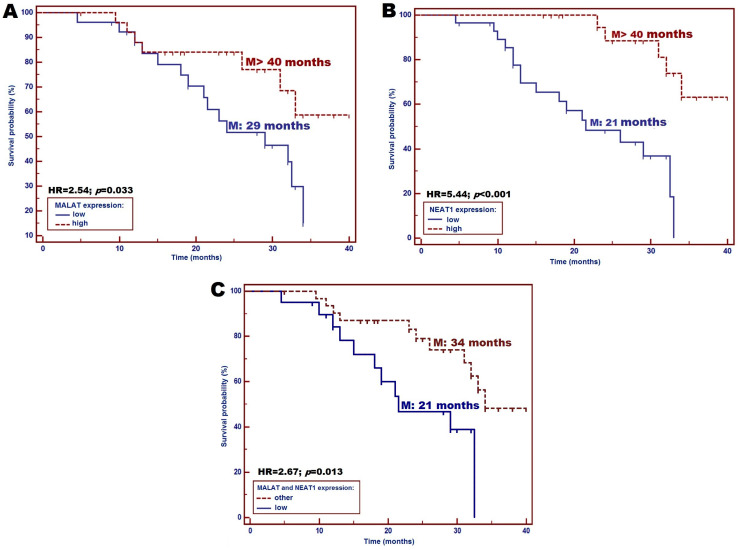
Impact of the studied lncRNAs on survival probability in the study group. (**A**) MALAT1; (**B**) NEAT1; (**C**) combined the both low MALAT1 and NEAT1 expression.

All patients enrolled in this study were followed up for 40 months. The incidence of death during the follow-up period was 37.1% (26/70 patients). The univariate survival analysis (Kaplan–Meier log-rank test) showed that patients with low MALAT1 expression had significantly shorter median OS compared to those with high MALAT1 expression (median OS: 29 months vs. >40 months; HR = 2.54; *p* = 0.033) (Figure 4A). Moreover, patients with low NEAT1 expression had more than a 5-fold higher risk of early death compared to other patients (median OS: 21 months vs. >40 months; HR = 5.44; *p* = 0.001) (Figure 4B). Additionally, the simultaneous presence of low MALAT1 and NEAT1 expression was significantly associated with about a 2.6-fold higher risk of OS reduction compared to patients with other expression variants (median OS: 21 months vs. 34 months; HR = 2.67; *p* = 0.013) (Figure 4C). The multivariate survival analysis (Cox proportional hazards model) revealed that low NEAT1 expression was an unfavorable independent factor affecting patient survival (HR = 5.88; *p* = 0.004).

## 4. Discussion

RT is a primary factor for LC but carries a high risk of adverse effects, including acute and late toxicities, observed in all HNC patients undergoing curative RT [9]. The early prediction of radiation toxicity is crucial for optimizing individualized treatment and improving RT planning [36]. Currently, routine biomarkers, such as biochemical and hematological parameters, are used to assess toxicity at various stages of treatment [37]. However, these markers are inadequate for the early detection and prediction of RT-related side effects.

LncRNAs in the bloodstream are emerging as diagnostic and prognostic markers for cancer due to their stability and role in disease progression [38]. They regulate genes and pathways influencing tumor response to RT, including miRNAs that modulate radiosensitivity [17]. Both miRNAs and lncRNAs are considered novel biomarkers for predicting clinical outcomes and RT-related side effects [39]. While recent studies have focused on miRNAs for HNC, markers like miR-9 and miR-21 are linked to RT response and toxicity [40]. miRNAs regulate responses to RT, including oxidative stress, DNA repair, and apoptosis [41]. It was confirmed that miR-200c is involved in radiation-induced oral mucositis in HNC patients [42]. miRNAs such as miR-1, miR-21, miR-133, miR-155, miR-208, and miR-221 have also been implicated in RT-associated cardiotoxicity [43]. LncRNAs like MALAT1 and NEAT1 contribute to radioresistance, with MALAT1 modulating resistance in nasopharyngeal cancer via miR-1 inactivation and NEAT1 promoting resistance through the miR-204/ZEB1 axis [44,45]. Recent studies have suggested that MALAT1 is widely upregulated in LC tissues compared to adjacent normal tissues [46]. However, the findings from different studies are not consistent. A significant decrease in the expression of MALAT1 was reported in the serum of HNC patients after IMRT (*p* < 0.001) [47]. Similarly, a decreased level of MALAT1 was noted in HNC patients after three cycles of CTH (*p* = 0.021) [48]. On the other hand, other studies observed higher MALAT1 expression in HNC patients (*p* < 0.001). High MALAT1 levels were significantly associated with clinical stage (*p* = 0.009), histological grade (*p* = 0.002), and lymph node metastasis (*p* = 0.011) in HNC patients [49]. In our study, we found only an association between MALAT1 expression and tumor grade (*p* = 0.040). Moreover, we observed a significantly lower expression of NEAT1 in stage III compared to stage IV according to the TNM classification (*p* = 0.027). Additionally, a lower NEAT1 expression was noted in T3-T4 tumors (*p* = 0.041) and in G3 tumors (*p* = 0.012) among the individuals studied.

There are no studies available that analyze the relationship between the expression levels of MALAT1 and NEAT1 and the development of RT-related adverse effects. The available literature has demonstrated relationships between single nucleotide polymorphisms (SNPs) in lncRNAs and the risk of toxic responses to treatment in cancer patients. For instance, it was confirmed that nasopharyngeal cancer patients undergoing chemotherapy with the CC genotype of lncRNA MEG3 had a significantly higher (threefold) probability of severe anemia (grades 3–4) (OR = 3.00; *p* = 0.007). In the same study, the GA genotype of pR-lncRNA-1 was associated with a higher chance of neutropenia (OR = 2.12; *p* = 0.047) [50]. Another study involving 467 individuals with lung cancer undergoing chemotherapy found that an SNP in lncRNA ANRIL was associated with a lower incidence of overall toxicity (grades 3–4) (*p* = 0.028). Additionally, the authors suggested that an SNP in MEG3 may be a valuable indicator for predicting the probability of severe gastrointestinal adverse effects caused by chemotherapy (*p* = 0.047). However, an SNP in MALAT1 was linked to a greater chance of severe gastrointestinal toxicity only in patients aged ≥57 years and smokers [51]. One study conducted in hepatocellular carcinoma patients confirmed a significant correlation between the expression of MALAT1 and levels of AST (*p* = 0.001) and hemoglobin concentration (*p* = 0.009) [52]. Our results are consistent with these findings, as we observed a higher frequency of anemia and liver failure in patients with a lower expression not only of MALAT1 but also of NEAT1. Both studied lncRNAs have been proposed as regulators of cancer-dependent inflammatory responses, targeting the STAT3 and NF-κB pathways and promoting the production of pro-inflammatory cytokines such as TNF-α [53].

In our study, we found a higher CRP concentration in patients with high MALAT1 and low NEAT1 expression levels after the completion of treatment, which supports the hypothesis that these lncRNAs play a pro-inflammatory role. MALAT1 has also been shown to regulate myogenesis, myogenic differentiation, and muscle regeneration through the modulation of MyoD transcription [54]. Additionally, MALAT1 has been associated with a low fat mass index (FMI) and poor prognosis in cancer patients, and it is underexpressed in the subcutaneous white adipose tissue of individuals with cancer-related cachexia [55]. In our study, both low MALAT1 (*p* < 0.001) and NEAT1 (*p* = 0.026) expression levels were associated with a higher probability of weight loss greater than 5% as a consequence of RT. Furthermore, a higher likelihood of BMI reduction after RT completion was linked to low MALAT1 expression (*p* = 0.044). Additionally, LC patients with low MALAT1 expression had a significantly higher chance of developing severe malnutrition (*p* < 0.001), while those with low NEAT1 expression had an increased risk of moderate and severe malnutrition (*p* = 0.002). Our study demonstrates that both low MALAT1 and NEAT1 expression levels are significantly associated with a higher probability of post-RT adverse effects, including anemia, liver failure, and nutritional deficiencies.

Investigating novel markers for predicting RT-related side effects is crucial, as they affect patients’ quality of life and OS. High MALAT1 expression is linked to poor survival in LC patients [49,56], while low NEAT1 expression correlates with longer OS in LC [57] and improved OS in HNC patients treated with RT and chemotherapy (CTH) [48]. Our study found that low MALAT1 and NEAT1 expression was associated with shorter OS in LC patients (median OS: 29 vs. >40 months for MALAT1, 21 vs. >40 months for NEAT1; *p* = 0.033 and *p* = 0.001, respectively). The combination of a low expression of both lncRNAs predicted poorer OS (median OS: 21 vs. 34 months; *p* = 0.013). Differences with other studies may result from variations in specimen types and treatment methods.

The implementation of MALAT1 and NEAT1 as novel molecular biomarkers in routine diagnostics could enhance diagnostic methods in personalized oncology. Plasma lncRNA analysis is a simple, robust, and reliable approach that uses non-invasive material collected during routine laboratory tests (liquid biopsy). The detection of lncRNAs, in combination with other blood parameters, can be used to assess the likelihood of toxic events before treatment, allowing for earlier intervention and prevention. These assays offer time- and cost-efficiency, as well as rapid detection, compared to other laboratory tests. Moreover, the analysis of the studied lncRNA expression demonstrates higher sensitivity and specificity in detecting the side effects of RT.

## 5. Conclusions

Differences in lncRNA expression between groups of LC patients with and without post-RT adverse effects suggest the potential use of these molecules as predictive and prognostic biomarkers. Our study has some limitations, including its retrospective design and the small sample size. However, the patients included are relatively homogeneous in terms of tumor stage and treatment type. Furthermore, the analysis of MALAT1 and NEAT1 expression demonstrates their clinical utility in predicting adverse effects in LC patients following RT-based therapy.

## Figures and Tables

**Table 1:** Baseline characteristics of the study group.

Factor		n = 70 (100%)
Gender	Male	58 (82.9%)
	Female	12 (17.1%)
Age	Mean (range)	64 (43–87)
	≥64 years	34 (48.6%)
	<64 years	36 (51.4%)
T stage	T1	3 (4.2%)
	T2	13 (18.6%)
	T3	24 (34.3%)
	T4	30 (42.9%)
N stage	N0	24 (34.3%)
	N1	9 (12.9%)
	N2	30 (42.9%)
	N3	7 (9.9%)
M stage	Mx	1 (1.4%)
	M0	52 (74.3%)
	M1	17 (24.3%)
Disease stage (TNM)	III	22 (31.4%)
	IVA	28 (40%)
	IVB	3 (4.2%)
	IVC	17 (24.4%)
Grading	G1	43 (61.4%)
	G2	15 (21.4%)
	G3	12 (17.2%)
Performance status (PS)	0	59 (84.3%)
	1	11 (15.7%)
Previous surgery	Yes	64 (91.4%)
	No	6 (8.6%)
Type of treatment	RT	37 (52.9%)
	C-RT	33 (47.1%)
Alcohol consumption	Yes	29 (41.4%)
	No	41 (58.6%)
Smoking status	Smoker	50 (71.4%)
	Non-smoker	20 (28.6%)
	Current smoker	46 (92%)
	Former smoker	4 (8%)
Subjective global assessment (SGA)	A	8 (11.4%)
	B	32 (45.7%)
	C	30 (42.9%)
Body mass index (BMI) [kg/m^2^]	median (range)	23.20 (14.53–34.37)
	≥23.20	53 (75.7%)
	<23.20	17 (24.3%)
Relative expression of MALAT1	median (range)	0.28 (0.02–0.64)
Relative expression of NEAT1	median (range)	0.38 (0.06–3.20)

BMI—body mass index; C-RT—chemoradiotherapy; M—metastatic spread; N—lymph node involvement; RT—radiotherapy; SGA—subjective global assessment; T—tumor site and size; TNM—tumor, node, and metastasis staging.

**Table 2:** Relationship between relative expression of the studied lncRNAs and tumor features.

Factor		Relative MALAT1 Expression		Relative NEAT1 Expression	
		Median (IQR)	*p*	Median (IQR)	*p*
T stage	T1+T2	0.45 (0.32–0.54)	0.446	0.43 (0.33–0.65)	0.041 *
	T3+T4	0.38 (0.27–0.53)		0.38 (0.28–0.46)	
N stage	N0+N1	0.39 (0.31–0.57)	0.840	0.39 (0.28–0.50)	0.861
	N2+N3	0.39 (0.27–0.53)		0.41 (0.30–0.46)	
Disease stage (TNM)	III	0.43 (0.31–0.55)	0.714	0.44 (0.33–0.56)	0.027 *
	IV	0.38 (0.27–0.53)		0.36 (0.28–0.46)	
Grading	G1+G2	0.43 (0.31–0.64)	0.040 *	0.42 (0.32–0.67)	0.012 *
	G3	0.33 (0.21–0.48)		0.31 (0.20–0.39)	

* Statistically significant results; IQR—interquartile range; TNM—tumor, node, and metastasis staging.

**Table 3:** Predictive value of the lncRNAs along with differences in their expression between groups of LC patients presenting different post-RT adverse events.

Factor		Relative MALAT1 Expression			Relative NEAT1 Expression		
		Median (IQR)	*p*	OR [95% CI] *p*	Median (IQR)	*p*	OR [95% CI] *p*
**RIOM**	1 and 2	0.38 (0.29–0.52)	0.891	2.45 [0.87–6.76]	0.39 (0.28–0.46)	0.522	3.29 [0.66–16.38]
	3	0.41 (0.29–0.55)		0.089	0.42 (0.29–0.53)		0.146
**Anemia**	Yes	0.31 (0.24–0.39)	0.023 *	5.36 [1.07–26.62]	0.30 (0.26–0.39)	0.008 *	5.26 [0.05–0.77]
	No	0.44 (0.30–0.64)		0.040 *	0.43 (0.32–0.49)		0.020 *
**Kidney failure**	Yes	0.32 (0.21–0.41)	0.567	1.62 [0.58–4.47]	0.34 (0.29–0.47)	0.344	0.87 [0.25–3.02]
	No	0.40 (0.23–0.54)		0.349	0.40 (0.31–0.61)		0.828
**Liver failure**	Yes	0.32 (0.22–0.33)	0.013 *	6.07 [1.11–33.05]	0.32 (0.25–0.41)	0.026 *	5.70 [1.38–23.45]
	No	0.47 (0.37–0.69)		0.037 *	0.44 (0.31–0.59)		0.016 *
**SGA**	A	0.28 (0.21–0.39)	0.753	1.21 [0.27–5.30]	0.29 (0.23–0.40)	0.212	13.09 [2.56–67.05]
	B or C	0.27 (0.21–0.36)		0.796	0.25 (0.18–0.29)		0.002 *
	A or B	0.31 (0.22–0.37)	0.036 *	9.75 [2.71–35.11]	0.26 (0.19–0.30)	0.042 *	4.67 [0.94–23.19]
	C	0.29 (0.22–0.44)		<0.001 *	0.22 (0.18–0.30)		0.059
**Weight loss 5%**	Yes	0.26 (0.20–0.35)	0.032 *	7.42 [2.46–22.44]	0.22 (0.18–0.28)	0.456	11 [1.33–98.81]
	No	0.31 (0.23–0.47)		<0.001 *	0.25 (0.19–0.32)		0.026 *
**Weight loss 10%**	Yes	0.19 (0.18–0.31)	0.015 *	2.27 [0.41–12.60]	0.22 (0.17–1.12)	0.034 *	1.15 [0.12–10.59]
	No	0.31 (0.23–0.38)		0.347	0.25 (0.18–0.27)		0.896
**BMI [kg/m^2^]** **before RT**	≥23.20	0.30 (0.21–0.37)	0.355	9.07 [2.30–35.72]	0.25 (0.20–0.28)	0.795	0.36 [0.09–1.35]
	<23.20	0.28 (0.19–0.40)		0.001 *	0.27 (0.18–0.35)		0.132
**BMI [kg/m^2^]** **after RT**	≥21.29	0.34 (0.26–0.39)	0.048 *	2.84 [1.02–7.89]	0.30 (0.28–0.31)	0.046 *	0.33 [0.62–15.26]
	<21.29	0.23 (0.18–0.37)		0.044 *	0.21 (0.17–0.28)		0.173
**CRP after RT [mg/L]**	≥5	0.28 (0.19–0.35)	0.852	4.91 [1.77–13.65]	0.23 (0.18–0.29)	0.419	6.15 [1.23–30.61]
	<5	0.27 (0.21–0.38)		0.002 *	0.26 (0.18–0.33)		0.026 *

* Statistically significant results. BMI—body mass index; CRP—C-reactive protein; CI—confidence interval; IQR—interquartile range; OR—odds ratio; RIOM—radiation-induced oral mucositis; RT—radiotherapy; SGA—subjective global assessment.

## Data Availability

The original contributions presented in this study are included in the article. Further inquiries can be directed to the corresponding author.
